# Acute appendicitis caused by metastatic adenocarcinoma from the lung: a case report

**DOI:** 10.1186/s40792-018-0467-7

**Published:** 2018-06-15

**Authors:** Yusuke Kimura, Takafumi Machimoto, Daiki Yasukawa, Yuki Aisu, Tomohide Hori

**Affiliations:** Department of Digestive Surgery, Tenri Hospital, 200 Mishima-cho, Tenri, Nara 632-8552 Japan

**Keywords:** Appendix, Metastasis, Lung cancer, Adenocarcinoma, Laparoscopic appendectomy, Emergency operation

## Abstract

**Background:**

Appendiceal metastasis from lung cancer is rare. However, it often causes acute appendicitis that requires emergency surgery. We herein report a thought-provoking case of appendiceal metastasis from lung cancer.

**Case presentation:**

A 71-year-old man was diagnosed with advanced lung cancer with multiple metastases and underwent chemotherapy. One month later, he developed acute appendicitis, and laparoscopic appendectomy was promptly performed. A swollen appendix and pus collection were observed during surgery. Histological analysis revealed an invasive adenocarcinoma in the appendix that infiltrated the mucosal, submucosal, and muscular layers. Positive immunostaining of thyroid transcription factor 1 indicated appendiceal metastasis of pulmonary adenocarcinoma, not a primary appendiceal malignancy. The postoperative course was uneventful, and the patient’s pulmonary internist resumed continuous chemotherapy after surgery.

**Conclusions:**

Although appendiceal metastasis from pulmonary adenocarcinoma is rare, it often results in acute appendicitis. Optimal therapy including emergency surgery should be performed without hesitation so that chemotherapy can be resumed as soon as possible.

## Background

Lung cancer (LC) is one of the most common cancers worldwide and the leading cause of death in Japan. Metastasis occurs in half of LCs, and the most common sites are the lymph nodes, adrenal glands, liver, bone, and brain [1]. Unusual metastatic sites, such as the small intestine and skin, have also been documented [2, 3]. However, appendiceal metastasis from LC is rare; it was reported in 1 of 2066 patients with LC [4]. Unfortunately, appendiceal metastasis often results in acute appendicitis (AA) that requires emergent surgery. Therefore, physicians must take this etiology into consideration because AA is a clinical diagnosis [5]. We herein report a thought-provoking case of appendiceal metastasis from LC.

## Case presentation

**Fig. 1:**
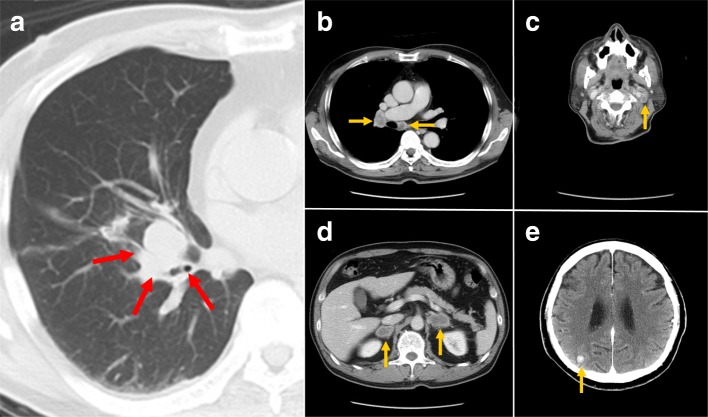
Far-advanced lung cancer on CT survey. **a** A primary lung tumor (red arrows) was located in the right middle lobe. Metastatic nodules (yellow arrows) were noted in the **b** lymph nodes at the hilum of the right lung and lymph nodes at the bifurcation of the trachea and **c** lymph nodes at the left side of the neck, **d** bilateral adrenal glands, and **e** brain

A 71-year-old man was referred to our hospital from his primary physician because of suspected LC. Computed tomography (CT) revealed a primary tumor in the right middle lobe and metastases in the lymph nodes (hilum of the right lung, bifurcation of the trachea, and left side of the neck), brain, both adrenal glands, and bones (Fig. 1). Pathological examination of a needle biopsy of the left cervical lymph node clearly revealed adenocarcinoma. Immunohistological findings showed positive staining of cytokeratin 7 and thyroid transcription factor 1 (TTF-1) and negative staining of cytokeratin 20. Therefore, we definitively diagnosed pulmonary adenocarcinoma and multiple metastases. His LC was categorized as stage IVB (T2a N3 M1c) according to the TNM classification [6].

**Fig. 2:**
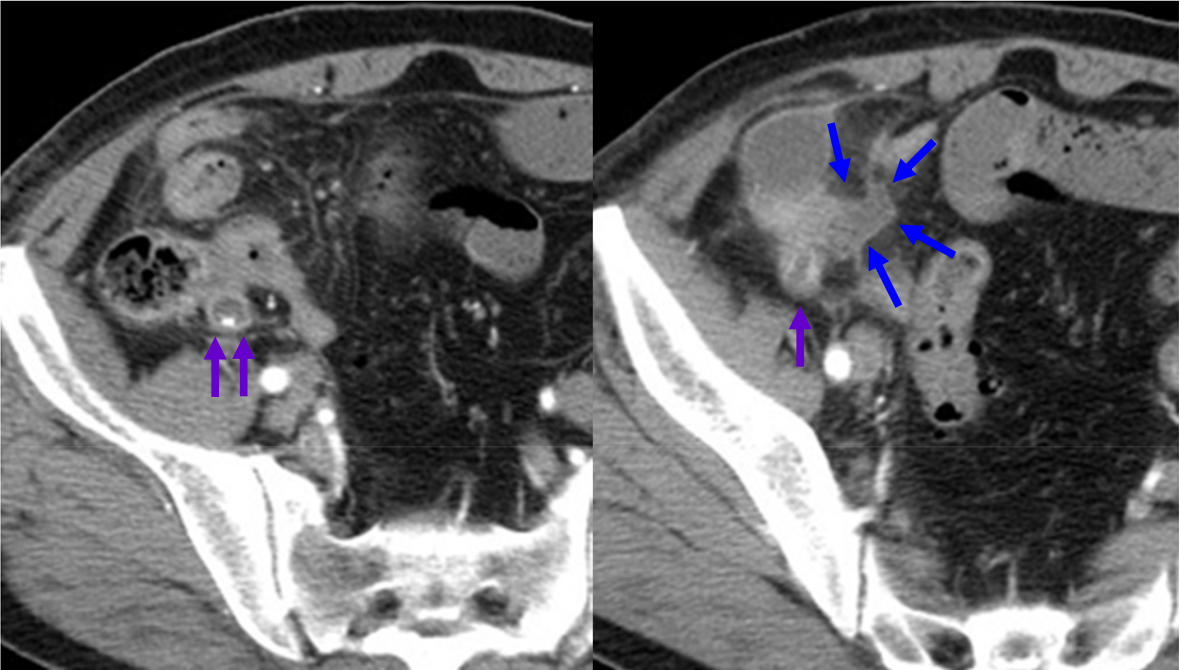
CT findings of acute appendicitis. A swollen appendix (purple arrows) was observed, and fluid collection (blue arrows) near the distal appendix was detected

**Fig. 3:**
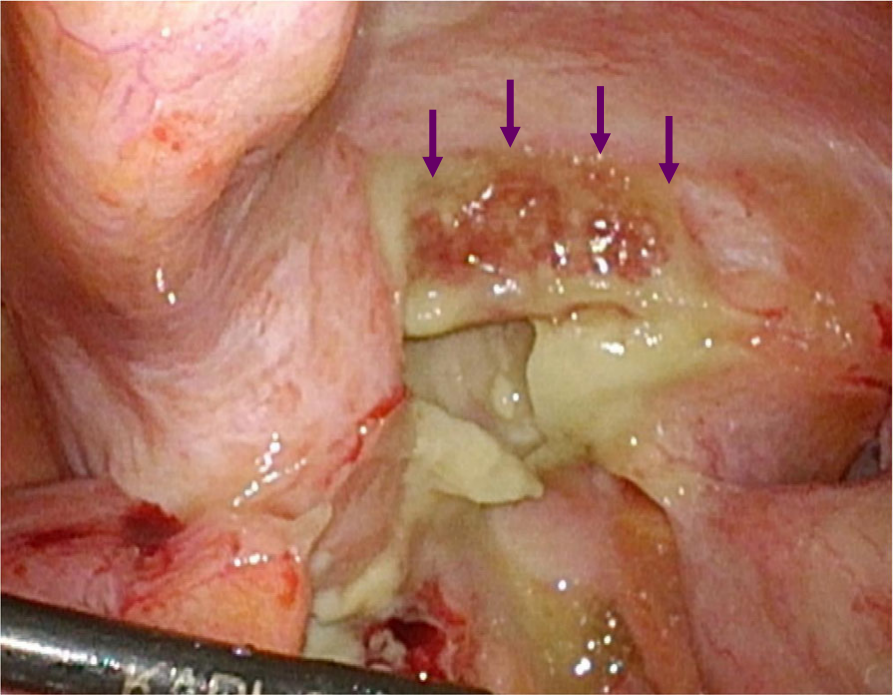
Laparoscopic findings. The appendix (purple arrows) was swollen, and pus collection was observed

This patient received chemotherapy with carboplatin, paclitaxel, and bevacizumab. One month later, he presented with right lower quadrant pain when he visited our hospital to receive his scheduled chemotherapy. His serum level of C-reactive protein was clearly increased at 11.67 mg/dL, although his white blood cell count was within the normal range. Enhanced CT findings showed an enlarged appendix and fluid collection near the distal appendix (Fig. 2). A diagnosis of AA was made, and laparoscopic appendectomy was promptly performed. A swollen appendix and pus collection were clearly observed during surgery (Fig. 3**)**. Laparoscopic survey of the abdominal cavity revealed no additional metastases (e.g., appendiceal tumor or peritoneal dissemination).

**Fig. 4:**
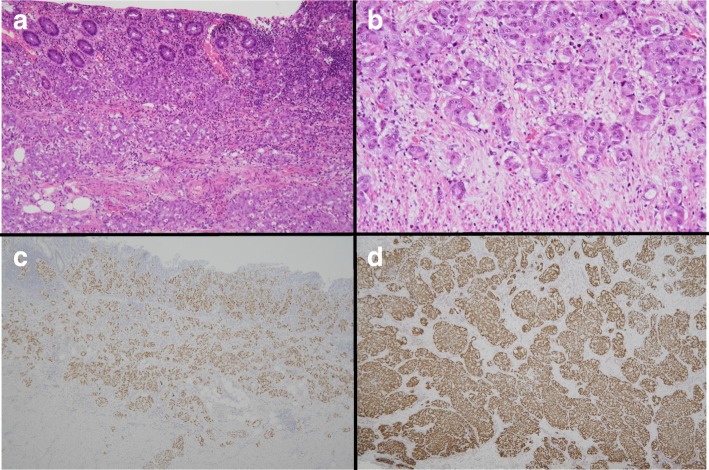
Pathological findings. The findings of hematoxylin and eosin staining are shown in **a** low-power field and **b** high-power field. Adenocarcinoma infiltrated the mucosal, submucosal, and muscular layers. Immunopathological findings of TTF-1 staining for the **c** appendix and **d** cervical lymph node are shown. The adenocarcinoma in the appendix and cervical lymph node showed positive staining for TTF-1

Histological analysis by hematoxylin eosin staining revealed invasive adenocarcinoma in the appendix that infiltrated the mucosal, submucosal, and muscular layers. Positive immunostaining of TTF-1 indicated that the appendiceal metastasis was from pulmonary adenocarcinoma, not a primary appendiceal malignancy (Fig. 4).

The postoperative course was uneventful, and the patient was discharged on postoperative day 7. The patient’s pulmonary internist resumed continuous chemotherapy after surgery.

## Conclusions

AA is a common disease worldwide, and primary appendiceal cancer often causes AA. However, secondary AA caused by appendiceal metastasis from LC is rare; the reported frequency of appendiceal metastasis of LC is 0.0005% [4]. With respect to the histological findings of LC that develops appendiceal metastasis, seven cases of small cell carcinoma [7–13] and four cases of adenocarcinoma [4, 14–16] have been reported. To the best of our knowledge, no other types of LC (e.g., squamous or large cell carcinoma) have reportedly caused appendiceal metastasis.

In our case, it was difficult to distinguish metastatic pulmonary adenocarcinoma from primary appendix cancer based only on the pathological findings of conventional hematoxylin and eosin staining. TTF-1 plays an important role in the immunopathological diagnosis of lung and thyroid cancers. Positive immunostaining of TTF-1 strongly indicates lung or thyroid cancer. In contrast, gastrointestinal and colorectal cancers generally show negative immunostaining of TTF-1, and only 2.5% of colon cancers are reportedly positive for TTF-1 [17]. Therefore, based on the immunopathological findings of TTF-1 immunostaining, we definitively diagnosed appendiceal metastasis from pulmonary adenocarcinoma, not a primary appendiceal cancer.

Although enhanced CT has an advantage in detecting metastatic masses in the appendix [15], many patients, including ours, show no appendiceal metastatic mass on imaging studies. Appendiceal metastases from LC are usually diagnosed only after AA has developed [7–14, 16], although fluorodeoxyglucose position emission tomography is reportedly useful for detection of appendiceal metastasis [12]. To the best of our knowledge, only one case of appendiceal metastasis without AA has been reported [15]. Hence, appendiceal metastasis from LC is usually diagnosed only after AA has developed. AA may worsen due to adverse effects of chemotherapy, and physicians must temporarily stop continuous chemotherapy for adequate treatment of AA. Accurate diagnosis of appendiceal metastases and subsequent optimal therapy including emergent appendectomy are important from the viewpoint of an effective therapeutic strategy for LC.

## Abbreviations

AA: Acute appendicitis
CT: Computed tomography
LC: Lung cancer
TTF-1: Thyroid transcription factor 1
